# Theoretical design and experimental verification of control system for building material packaging unit based on risk management

**DOI:** 10.1038/s41598-023-51102-3

**Published:** 2024-01-03

**Authors:** Jiayu Fang, Bin Deng

**Affiliations:** 1 University of Sydney, 21 Ross Street, Forest Lodge, Sydney, Australia; 2 Tianping College of Suzhou University of Science and Technology, No. 55 Changjiang Road, Suzhou, China

**Keywords:** Engineering, Civil engineering

## Abstract

Facing the shortage of special building materials packaging machinery with thermal insulation and low intelligence, this paper designs a set of mechanical and electrical integration packaging unit control system to reduce the risk of material transportation for different stakeholders. According to risk management tools, the system takes Mitsubishi PLC as the control core and combines with communication module, servo motor drive system and touch screen man–machine interface to realize the risk simulation and automatic control of the packaging unit. The simulation results of PID control model show that the parameters such as speed and torque can be stabilized in a relatively short period of time when the load is suddenly changed within 1.5 s. Theoretical verification of the system has small steady-state error, rapid response, and good control effect. The man–machine interface design was carried out and the actual corresponding test experiment was carried out. The experimental results showed that the overall operation rate of the packaging unit system reached 98.15%, the pass rate was 99.03%, and the production capacity was about 9600 packs/hour, which met the production requirements. The control system of the building material packaging unit designed in this paper realizes the equipment intelligence, has a high degree of automation, and shows good potential application value in the aspects of building information, reduction of construction risks and manufacturing intelligence.

## Introduction

With the development of Germany’s Industry 5.0 and the concept of risk management, a wave of smart manufacturing technology that combines production equipment, systems and smart terminals through the Internet has swept across the world, showing great potential for application in the fields of industry, agriculture, healthcare, materials, and commerce^1–3^. The research of intelligent manufacturing technology focuses on the deep integration of next-generation risk management and information technology, such as blockchain and Internet of Things (IoT), with industrial systems to realize the security, digitization, networking and intelligence of production equipment^4^. It is necessary for the upgrading and development of manufacturing industry^5^.

The infrastructure industry is the mainstay of economic and social development in various countries, and building materials play a vital role in the intelligent manufacturing of new infrastructure^6^. Zhou et al*.* discussed the performance of ferroelectric materials, cement building materials, magnesium oxide structural insulation panels in buildings from the perspective of efficiency and benefit, aiming to provide constructive suggestions for smart manufacturing in smart cities^7–9^. Kazi et al*.* developed intelligent manufacturing methods and models focusing on Industry 5.0 to predict the load and displacement curve characteristics of composite materials such as cotton fiber and ceramics^10,11^. However, traditional transportation and packaging of building materials mainly rely on manual packing, which has the disadvantages of difficult operation, high labor intensity, high cost and low degree of automation^12,13^. Especially for green thermal insulation materials such as expanded polystyrene, phenolic foam and styrofoam, there are certain risks due to their flammability and the generation of toxic gases when burned^14–16^. Therefore, the packaging and monitoring automation of green thermal insulation materials is very important for the control and decrease of building construction risks.

In recent years, the packaging and automation of building materials have gradually received widespread attention from researchers^15–19^. In 2021, Davydov et al*.* developed a pneumatic unloader and proposed a lifting device to unload and transport building materials to the destination through transportation and lifting, and the results showed that the device can effectively improve the transportation efficiency^17^. In 2022, Liu et al*.* explored the impact of macro-encapsulation and micro-encapsulation on the encapsulation of building materials, and aimed at the corresponding simulation and model building methods for different packaging methods^18^. Khramov et al*.* verified that the systematic combination of leather and belt conveyors can efficiently realize the transportation of building materials and the removal of waste materials through theory and experiments^19^. However, there are still few related studies on the automation of control and monitoring of packaging and conveying systems, especially experimental studies on packaging systems. In addition, current research on risk issues in building materials mainly focuses on the safety risks of the materials themselves^20–22^ and project construction risks^23–25^, the risk issues in the packaging and transportation system have hardly been paid attention to. Therefore, the development of a highly automated building materials packaging control system based on the risk issues of packaging and transportation systems, is of great significance to the safety and efficiency of building material construction and the development of intelligent manufacturing technology^26,27^.

This article intends to conduct risk assessment based on different stakeholders and risk management theories, and develop a control system based on PLC control and AC servo system drive to overcome the problems of high labor intensity and low production efficiency of manual packaging of special building materials. By establishing PID control and servo control system models, parameters such as speed and position are optimized, and on-site experiments on building materials packaging are carried out to verify the anti-interference ability, steady-state accuracy and packaging efficiency of the control system.

## Risk assessment-based design of a unit control system for packaging construction materials

### Risk assessment of construction material packaging unit

Sodangi et al*.* shows that operator risks and equipment management risks affect the safety of packaging unit in 2023^28^. Regarding safety monitoring risks and equipment maintenance risks, Zscheischler et al*.* argues that risk management should be strengthened, especially in the case of machinery operation^29,30^. The construction material packaging unit is different from the traditional packaging unit, which couples the LEC evaluation and risk index matrix in the study. We complete the evaluation of construction material packaging unit based on the conditional value-at-risk (CVaR) model in this study.

**Figure 1:**
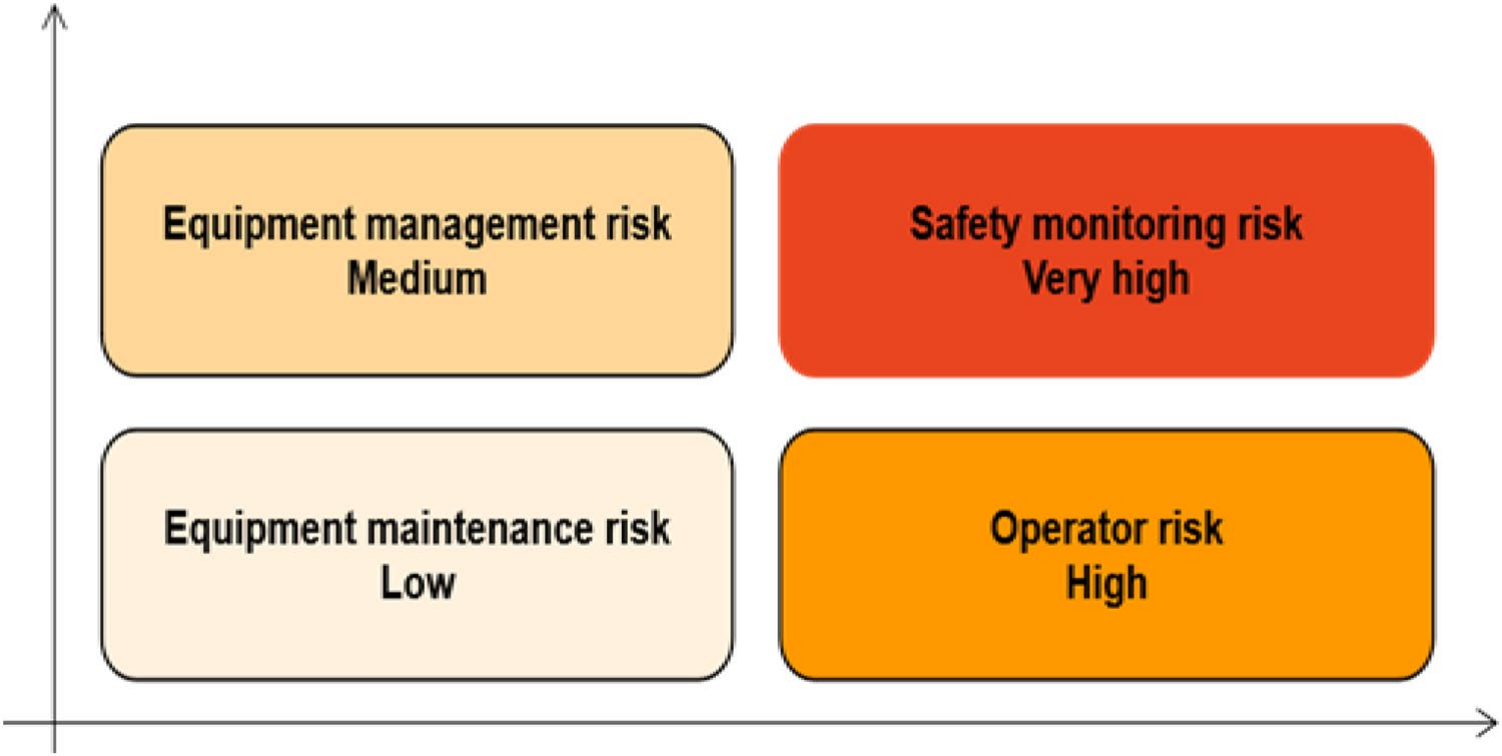
Risk index matrix coupling. Notes: Red is very high risk.

**Figure 2:**
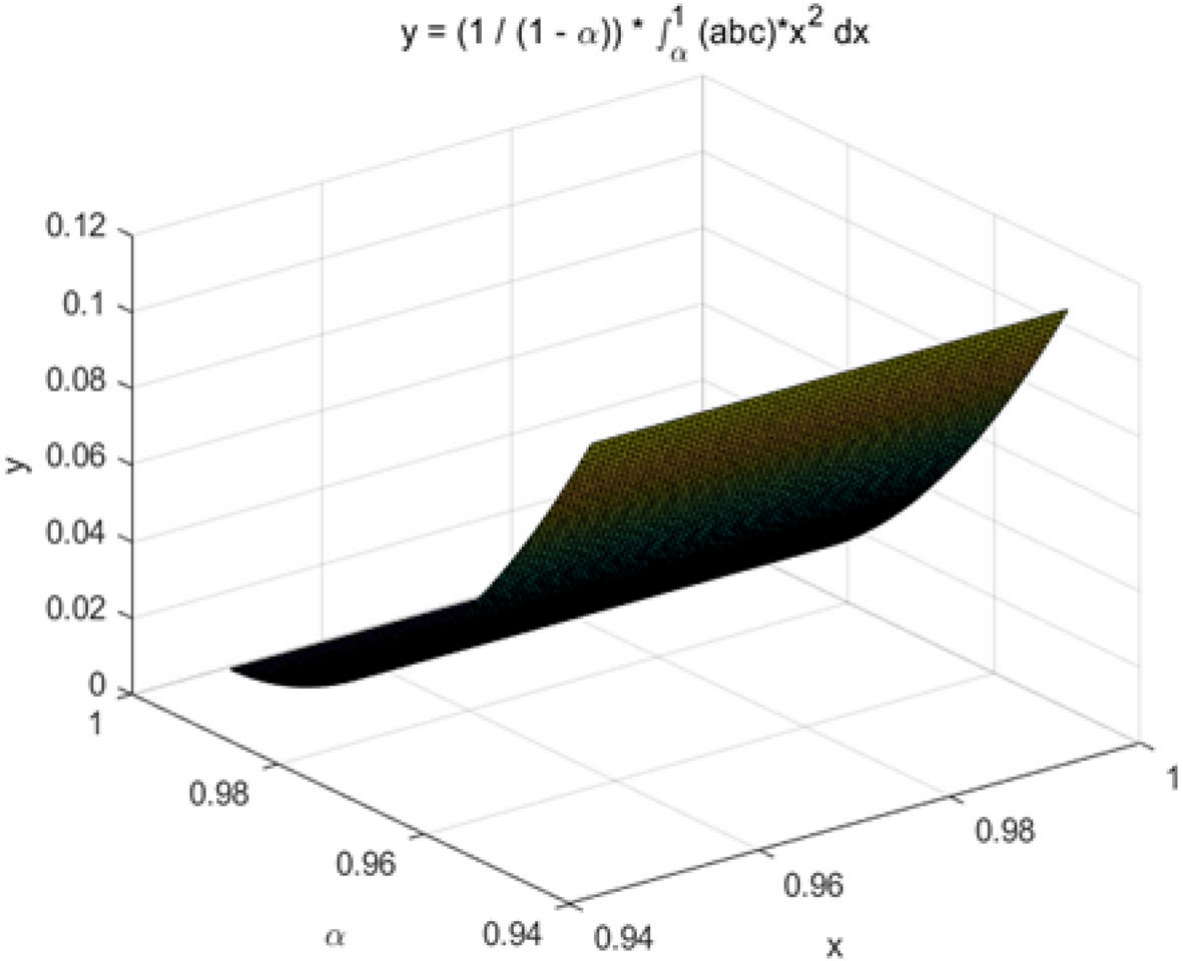
The model of CVar. Notes: CVar = (1 / (1–α)) $\times$∫[α, 1] f(x) * x dx^37^.

Risk identification: Risk identification uses analytical tools to quickly find the key factors to capture the focus of risk management. Because there are few actual projects on construction material packaging unit, this paper uses literature analysis and Risk Breakdown Structure (RBS) to calculate risk probabilities (Table 1). Consequently, this paper will complete the risk evaluation and simulation from the perspectives of operator risk, equipment management risk, safety monitoring risk, and equipment maintenance risk.
(2) Risk evaluation: Different risk factors are quantified and analyzed in a risk evaluation. This approach helps stakeholders to develop targeted risk management measures. The risk factors are quantitatively analyzed by applying LEC evaluation (Eq. 1)^35^ through the risk influencing factors mentioned above. In addition, the likelihood of accidents (L), the frequency of human exposure to material packaging machinery (E), and the loss consequences of the occurrence of risk (C) will be referred to Table 2 (C: four-level risk classification).
  1 $$D=L\times E\times C$$

where $D$ and $L$ represent the degree of danger and the size of the possibility of accidents, $E$ and $C$ refers to the frequency of human exposure to material packaging machinery, respectively.Risk simulation: the risk simulation of construction material packaging unit involves the above LEC evaluation and risk index matrix coupling. Figure 1 shows that the safety monitoring risk of construction material packaging machinery is high risk. Meanwhile, Conditional Value at Risk (CVar)^36^ is simulated based on the standard deviation of the normal distribution. The average loss value (ALVR) of CVar at risk level α is shown in Fig. 2, and the color of ALVR is from dark to light. In the case of exceeding the risk level α, ALVR can help decision makers to identify potential risks at higher α. This part simulates the range of risk evaluation and the size of CVar thus providing the basis for the simulation of the PID risk control model.

**Table 1:** Key risk influencing factors for material packaging unit in Web of Science, 2022.

Factors influencing	Total Probability	RBS and probability	References of RBS
Operator risk	0.10	Environment 0.10 Materials < 0	Cheng et al (2014)^31^
Equipment management risk	0.035	Maintenance 0.035 Aging < 0	Idoniboyeobu et al. (2018)^32^
Safety monitoring risk	1.3	Technology 0.36 Data 0.47 Privacy 0.035	Nnaji et al (2021)^33^
Equipment maintenance risk	< 0	Frequency < 0 Timeliness < 0	Jafarpisheh et al. (2021)^34^

Probability = Number of papers/Total number papers, TotalProbability = p_a_ + p_b_ + p_c_.

**Table 2:** Four-level risk classification in material packaging unit.

Factors influencing	Low (1)	Medium (2)	High (3)	Very high (4)
Operator risk		✓		
Equipment management risk			✓	
Safety monitoring risk				✓
Equipment maintenance risk	✓			

Order of probability according to Table 1, source from Baybutt, (2018)^30^.

### Construction material packaging unit

**Figure 3:**
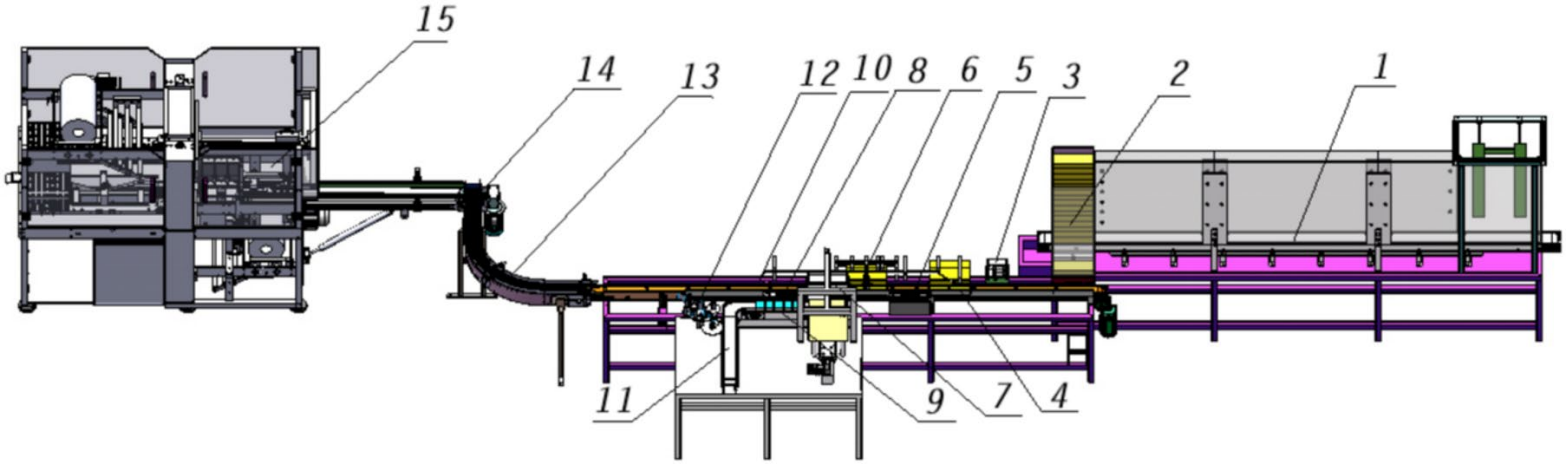
Schematic diagram of the overall 3D model of the building material packaging unit (1. Conveying empty carton channel 2. Magazine type storage bin 3. Box opening mechanism 4. Empty carton 5. Paper turning mechanism 6. Rope spreading mechanism 7. Cartoning mechanism 8. Paper covering mechanism 9. Hard box 10. Closing mechanism 11. Hard box input conveyor belt 12. Labeling mechanism 13. 90° chain plate conveyor 14. Turning push mechanism 15. Outer film packaging machine).

Based on the above-mentioned risk management of equipment management risk, safety monitoring risk and operational risk, the design of building materials packaging unit corresponds to one of the three stages, that is the preparation stage (equipment management), working stage (safety monitoring) and completion stage (operational risk). The packaging object of the hard-core packaging unit for building materials is special thermal insulation building materials. The packaging unit mainly consists of a carton comprehensive packaging machine, a transparent paper packaging machine and a conveyor, as shown in Fig. 3. The packaging process is mainly divided into three parts: boxing, labeling and outer film packaging.

1. Preparation stage: Qualified building materials are transported from the transparent paper packaging machine to the carton packaging machine channel (1 in Fig. 3). The sensor detects that the building materials are in place and starts the stacking task. The small boxes are formed into two rows of five bags of small boxes side by side through the cylinder lifting and loading mechanism. At the same time, the carton conveyor transports the printed empty cartons from the clamp-type storage bin (2 in Fig. 3) to the main conveyor belt.
2. Working stage: The paper turning process is carried out by the paper turning mechanism (5 in Fig. 3) between the unpacking mechanism (3 in Fig. 3) and the rope tensioning mechanism (6 in Fig. 3). First open the box cover and sulfuric acid paper, and then start packaging the building materials after the photoelectric switch detects that the empty carton (4 in Fig. 3) is in place. The cylinder piston presses into the small carton (9 in Fig. 3) and passes through the cartoning mechanism (7 in Fig. 3), paper covering mechanism (8 in Fig. 3), closing mechanism (10 in Fig. 3), and labeling mechanism (12 in Fig. 3) for processing, and then sent to the box outer film packaging machine by the chain conveyor (13 in Fig. 3).
3. Completion stage: The building material boxes arrive at the pushing mechanism (14 in Fig. 3), and after photoelectric detection, the outer film packaging machine (15 in Fig. 3) is started. The box conveyor belt and various mechanisms work in regular cycles. The building material boxes are delivered to the jacking station, wrapped with outer film, and both ends are hot-melt sealed to complete all packaging.

### Design of control system of packing unit

**Figure 4:**
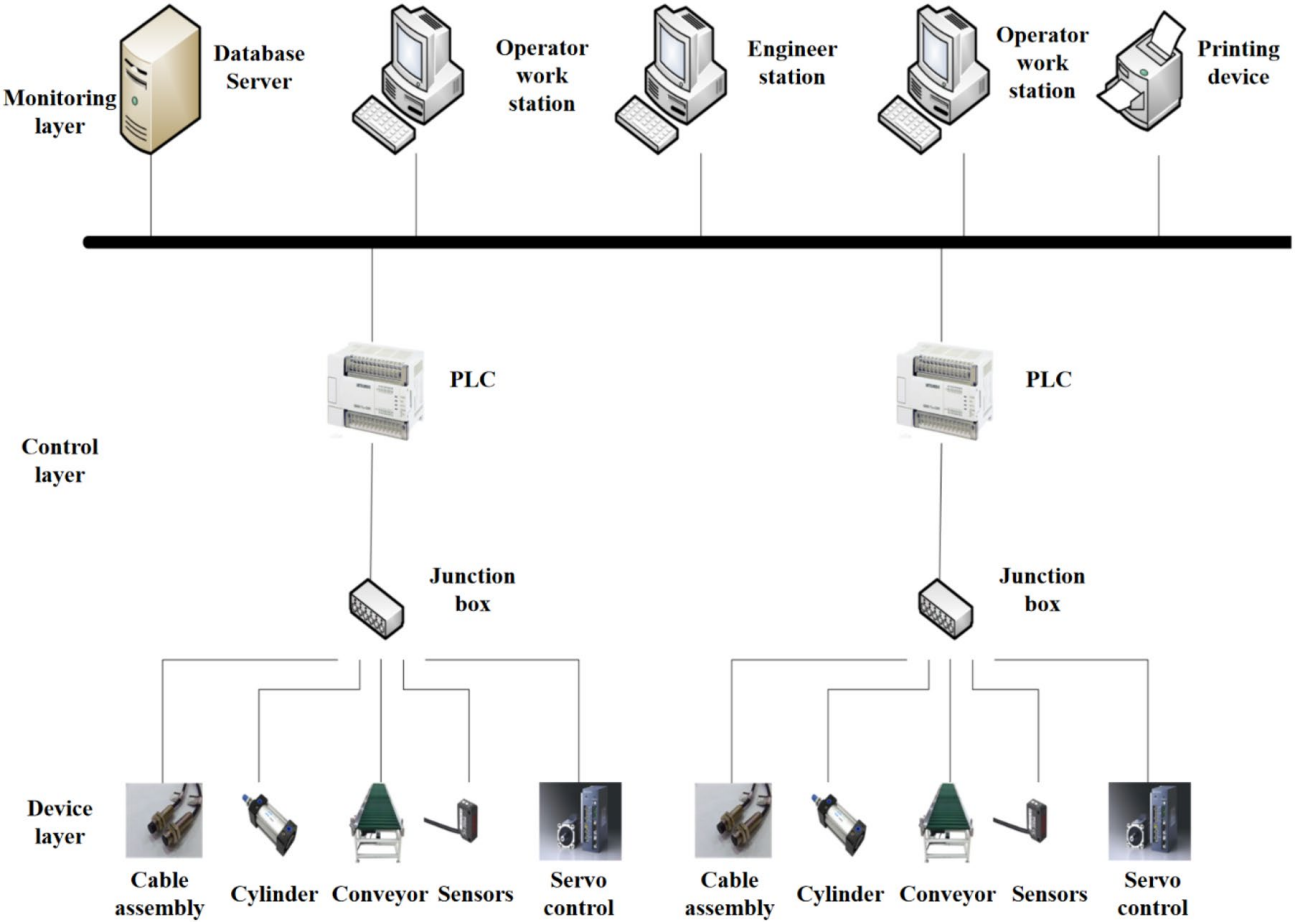
Schematic diagram of the network structure of the control system.

The main task of the construction material packaging unit is to complete the three links of boxing, labeling and outer film packaging, but each link includes multiple processes. Therefore, several control units must cooperate and coordinate with each other, which puts forward certain requirements for the intelligence of the control system. The realization principle of the intelligent control system is to form a complex system through the Internet to form the monitoring layer, control layer and equipment. The network topology of the control system is shown in Fig. 4.

From the perspective of the equipment layer, the CAN-based fieldbus technology^38–40^ can not only realize the information exchange between the underlying equipment and the controller, but also can be directly connected through a cable without the I/O module interface. It is also free to access or move devices without disconnecting from network power. In the control layer, N:N network connections can be used between PLCs, and smart devices, such as computer interfaces, can be connected to PLC processors and I/O ports.

The upper computer system such as touch screen or industrial computer can directly manipulate the lower computer. The monitoring layer embeds the TCP/IP protocol in the Ethernet processor, and the Ethernet is directly connected to the processor without special modules and network components.

The main controller of the packaging unit is PLC, which is responsible for the operation and control of labeling machine, conveyor, servo motor, frequency converter and vacuum pump. The information collection of the underlying equipment on site is mainly completed by various sensors, including 14 photoelectric switches, 4 proximity switches, and 1 pressure detection switch. There are 3 manual operation buttons for start, stop and emergency stop, and 2 manual/automatic work mode switching switches. During the speed regulation process of the frequency converter, it is necessary to check whether the running state is good or not, which occupies two input points. The status indicator lights are set to power on, normal operation, and fault alarm, which can clearly see the working status of the system. To sum up, a total of 22 digital input signals and 22 digital outputs are required. According to the working characteristics of the construction material hard box packaging unit, Mitsubishi FX3U-48MR PLC is selected, and a positioning module is added to meet the needs of positioning control in equipment production. The I/O address assignment of the control system interface is shown in Table 3.

**Table 3:** I/O address assignment of control system interface.

Function description	Symbol	Port Address	Function description	Symbol	Port Address
Stacking in place detection	SQ1	X0	Vacuum Pump	KM1	Y0
Strip box level detection	SQ2	X1	Servo Motor	KM2	Y1
Label level detection	SQ3	X2	Inverter start/stop	KM3	Y2
pump pressure detection	SQ4	X3	Small box stacking	YV1	Y3
Push-in-position detection	SQ5	X4	Push in the box	YV2	Y4
Clamping in place detection	SQ6	X5	Clamp the box	YV3	Y5
Lid pick-up detection	SQ7	X6	Open the lid	YV4	Y6
Strip box position detection	SQ8	X7	Turn over sulfate paper	YV5	Y7
Drawstring grip detection	SQ9	X10	Unfold the drawstring	YV6	Y10
Air jaw release detection	SQ10	X11	Put in the box	YV7	Y11
Pressing in place detection	SQ11	X12	Cover with sulfate paper	YV8	Y12
Labeling in place detection	SQ12	X13	Close the lid	YV9	Y13
Labeling completion detection	SQ13	X14	Labeling	YV10	Y14
Turning conveyor detection	SQ14	X15	Conveyor	YV11	Y15
Heat seal completion detection	SQ17	X16	Topping up	YV12	Y16
Inverter operation normal	SQ18	X17	Cut off outer film	YV13	Y17
Inverter failure	SQ19	X20	Heat seal the outer film	YV14	Y20
Stop button	SB1	X21	Missing label alarm	YV15	Y21
Start button	SB2	X22	bin level is low	YV16	Y22
Emergency stop button	SB3	X23	Power on	HL1	Y23
Automatic mode	SB4	X24	Normal operation	HL2	Y24
Manual mode	SB5	X25	Fault alarm	HL3	Y25
Stacking in place detection	SQ1	X0	Push in the box	KM1	Y0
Strip box level detection	SQ2	X1	Clamp the box	KM2	Y1

Combined with the technological process and control requirements of the building material hard box packaging unit, and through the analysis of the action sequence between the various actuators, the program design of the PLC software control is mainly composed of a main program and multiple subroutines. When the packaging unit is working, it needs to control a variety of operating signals. The main program is mainly used to process logic signals such as start, stop and emergency stop, including the signal of each station in place, and various initialization default value signals before the unit starts, the selection signal of automatic or manual working mode, and multiple reset signals, etc.

**Figure 5:**
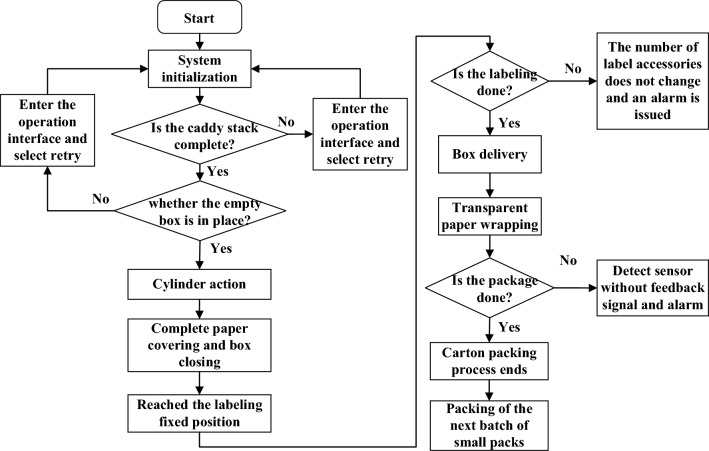
Control flow chart of building material packaging unit.

Subroutines include manual subroutines, automatic subroutines, alarm subroutines and historical data report subroutines, etc., which are responsible for processing commands generated by human–computer interaction, and finally realize the function of automatic packaging production of building material hard box units. Algorithm implementation of control flow is shown in Fig. 5.

### Control function model of automatic packaging production line

The main controller of the packaging unit is PLC, which is responsible for the operation and control of servo motors, conveyors, frequency converters, vacuum pumps, etc. The principles and mathematical models involved in each part are introduced in detail in this section.

### Control function *model* of automatic packaging production line

Modern AC servo systems include servo motors, power converters, controllers, detectors, etc.^41^. When designing the control system of the packaging unit, it is necessary to ensure that the equipment can run stably with a certain accuracy, and at the same time, there is no large speed fluctuation under various disturbances, so as to ensure that the product packaging is free from damage and the external surface is smooth and smooth. This is the case in the field of industrial automation. PID control algorithm is usually used for closed-loop control problems. When the target model is determined and the working environment and operation steps are basically unchanged, the PID control strategy is effective and has been widely used in dynamic control.

**Figure 6:**
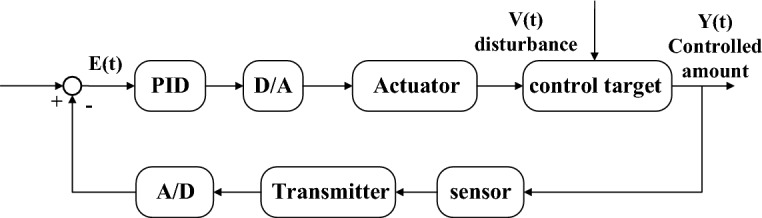
PID closed-loop control structure block diagram.

The control method adopted by the PID algorithm is as follows: in the control system of the packaging unit, the PLC compares the target values of various parameters with the actual values measured by the sensors and transmitters, and uses the PID calculation to obtain the frequency converter and Operating control value of the servo drive. The inverter can output the power supply voltage corresponding to the frequency to control the various actuators of the packaging unit. By adjusting parameters such as torque, speed and pressure, and then modulating the pulse width of the solid-state relay, the power supply voltage of the servo drive is controlled. The adjusted position parameter reaches a stable value, and the control structure diagram is shown in Fig. 6. Before using the PID operation command, the given value, measured value and control parameters must be set and adjusted. Among them, the given value is the system demand value set on the touch screen, the actual value in the production process is measured by the sensor, the actual value is generated by the feedback of the transmitter and the A/D module, and the final value of the control parameter is related to PID operation.

Each part of the packaging machine of the hard box packaging unit for building materials is driven by a separate motor, and the main shaft is connected to the motor to drive the actuator. Therefore, the coordinated control of the speed and distance of each part of the actuator can be realized by controlling the motor speed and torque control. To achieve high-performance speed regulation, it is necessary to introduce the model^42^. When studying its mathematical model, it is usually considered that its magnetic circuit is linear in an ideal state, the iron core has no loss, and the simplified model in the d-q coordinate system is established without considering the frequency change and harmonic influence.

Stator voltage equation:

2 $$ \left\{ \begin{gathered} uds = Rsids + p\Psi ds - we\Psi qs \hfill \\ uqs = Rsiqs + p\Psi qs - we\Psi ds \hfill \\ \end{gathered} \right. $$

Rotor voltage equation:

3 $$ \left\{ \begin{gathered} udr = Rridr + p\Psi dr - we\Psi qr \hfill \\ uqr = Rriqr + p\Psi qr - we\Psi dr \hfill \\ \end{gathered} \right. $$

Stator flux equation:

4 $$ \left\{ \begin{gathered} \Psi ds = Lsids + Lmidr \hfill \\ \Psi qs = Lsiqs + Lmiqr \hfill \\ \end{gathered} \right. $$

Rotor flux equation:

5 $$ \left\{ \begin{gathered} \Psi dr = Lmids + Lsidr \hfill \\ \Psi qr = Lmiqs + Lsiqr \hfill \\ \end{gathered} \right. $$

Electromagnetic torque equation:

6 $$ Te = pnLm(iqsidr - iqrids) $$

where ${R}_{S}$ and ${R}_{r}$ are the internal stator and rotor resistances of the asynchronous motor, respectively. ${L}_{S},$ ${L}_{r}$ and ${L}_{m}$ are the equivalent self-inductance and mutual inductance of the stator and rotor inside the asynchronous motor, respectively. ${u}_{ds}, {u}_{qs}, {u}_{dr}, {u}_{qr}$ are respectively stator and rotor voltage $d, q$ axis components. ${i}_{ds},{i}_{qs}, {i}_{dr}, {i}_{qr}$ are the stator and rotor current $d, q$ axis components respectively. ${\Psi }_{ds}, {\Psi }_{qs}, {\Psi }_{dr}$ and ${\Psi }_{qr}$ are respectively the stator and rotor flux $d, q$ axis components. ${w}_{e}$ is the angular velocity, and ${p}_{n}$ is the pole logarithm.

The vector control strategy mainly takes decoupling control as the core, so the coordinate transformation of the current is carried out. Three-phase-two-phase stationary coordinate transformation:

7 $$ \left[ \begin{gathered} i\alpha \hfill \\ i\beta \hfill \\ \end{gathered} \right] = \sqrt{\frac{2}{3}}  \left[ \begin{gathered} 1 - \frac{1}{2} - \frac{1}{2} \hfill \\ 0\frac{\sqrt 3 }{2} - \frac{\sqrt 3 }{2} \hfill \\ \end{gathered} \right]\left[ \begin{gathered} ia \hfill \\ ib \hfill \\ ic \hfill \\ \end{gathered} \right] $$

Two-phase stationary-rotating coordinate transformation:

8 $$ \left[ \begin{gathered} id \hfill \\ iq \hfill \\ \end{gathered} \right] = \left[ \begin{gathered} \cos \theta \sin \theta \hfill \\ - \sin \theta \cos \theta \hfill \\ \end{gathered} \right]\left[ \begin{gathered} i\alpha \hfill \\ i\beta \hfill \\ \end{gathered} \right] $$

Two-phase rotation-stationary coordinate transformation:

9 $$ \left[ \begin{gathered} i\alpha \hfill \\ i\beta \hfill \\ \end{gathered} \right] = \left[ \begin{gathered} \cos \theta - \sin \theta \hfill \\ \sin \theta \cos \theta \hfill \\ \end{gathered} \right]\left[ \begin{gathered} id \hfill \\ iq \hfill \\ \end{gathered} \right] $$

### Conveyor *control* model

According to the work content, the conveyor belt in the production line can be divided into two parts: the robot carrying end conveyor belt, the through hole and the packing section conveyor belt. According to the composition of the conveyor belt, the above two types of conveyor belts both use chain drive^43^. The current conveyor belt speed can be calculated using Eq. (10).

10 $$v=\frac{{z}_{1}p{n}_{1}}{60\times 1000}=\frac{{z}_{2}p{n}_{2}}{60\times 1000}$$

where $p$ represents the side length of the conveyor belt, $z$ represents the number of sensors, and the variables ${n}_{1}$ and ${n}_{2}$ are the rotational speeds of two single sprockets respectively.

It can be seen that during the operation of the conveyor belt, the speed of conveyor belt can be controlled by controlling the rotation speed of the two sprockets. During the speed control process, the impact of the weight of objects on the surface of the conveyor belt on the speed needs to be considered to ensure speed control accuracy. If the displacement resistor signal changes continuously, the change value of the signal is directly used to drive the brake, thereby adjusting the braking torque in the controller to keep the tension constant. In this way, automatic adjustment of torque and constant tension control of packaging can be achieved. The control of constant tension follows the physical principle of rigid body rotation, and its expression is:

11 $$F=\frac{M}{d}$$

where $M$ is the moment, $d$ is the moment arm that controls the rotating axis, and $F$ is the acting force. In the actual packaging and unloading process, the value of $d$ can be the radius of the packaging box.

### Control model of *packaging* volume and frequency

The setting of the system software program communication transmission clock determines the frequency of system control task execution. The input clock frequency of each timer can be expressed as:

12 $$f=\frac{{f}_{PC}}{{f}_{TC1}\left({f}_{TC0}+1\right)}$$

where ${f}_{PC}$, ${f}_{TC0}$ and ${f}_{TC1}$ represent the controller bus, configuration register and frequency division value of the timer, respectively. According to the above method, according to the designed production line process operation mode, the setting of the communication transmission clock frequency of the control equipment and mechanical equipment is completed in sequence.

The calculation program of the production line control volume mainly occurs in the architecture controller. The real-time signal detection results of the sensor are input into the controller, and the operating parameters of each mechanical equipment in the actual packaging production line are set. From this, Eq. (13) can be used Calculate the control volume of the production line.

12 $$N=\left|{n}_{0}-{n}_{i}\right|$$

where ${n}_{0}$ and ${n}_{i}$ are the operating parameter setting values and detection values of any mechanical equipment in the production line. The calculation result of Eq. (12) is used as the control instruction output by the controller and distributed to each production line mechanical equipment.

## Results and discussions

### Performance simulation verification of packaging unit control system

**Figure 7:**
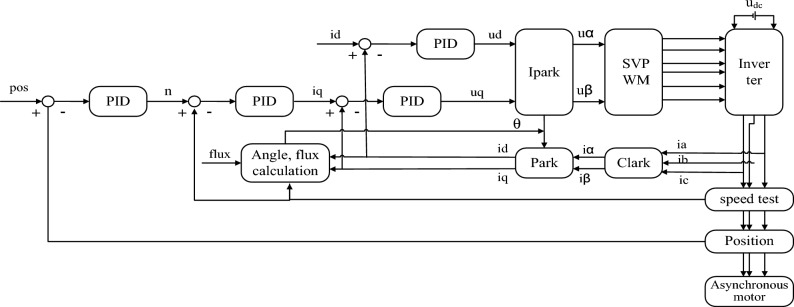
Structural diagram of servo control system based on PID controller.

In order to further verify the performance of the control system of the packaging unit, according to the above-mentioned analysis and modeling of the servo drive control system, a PID control simulation model is designed on the MATLAB/Simulink platform, and the simulation analysis is carried out. It can be seen from Fig. 7 that the control architecture mainly includes four parts: position loop PID regulator, speed loop PID regulator, current loop PID regulator, SPWM algorithm, etc.

**Figure 8:**
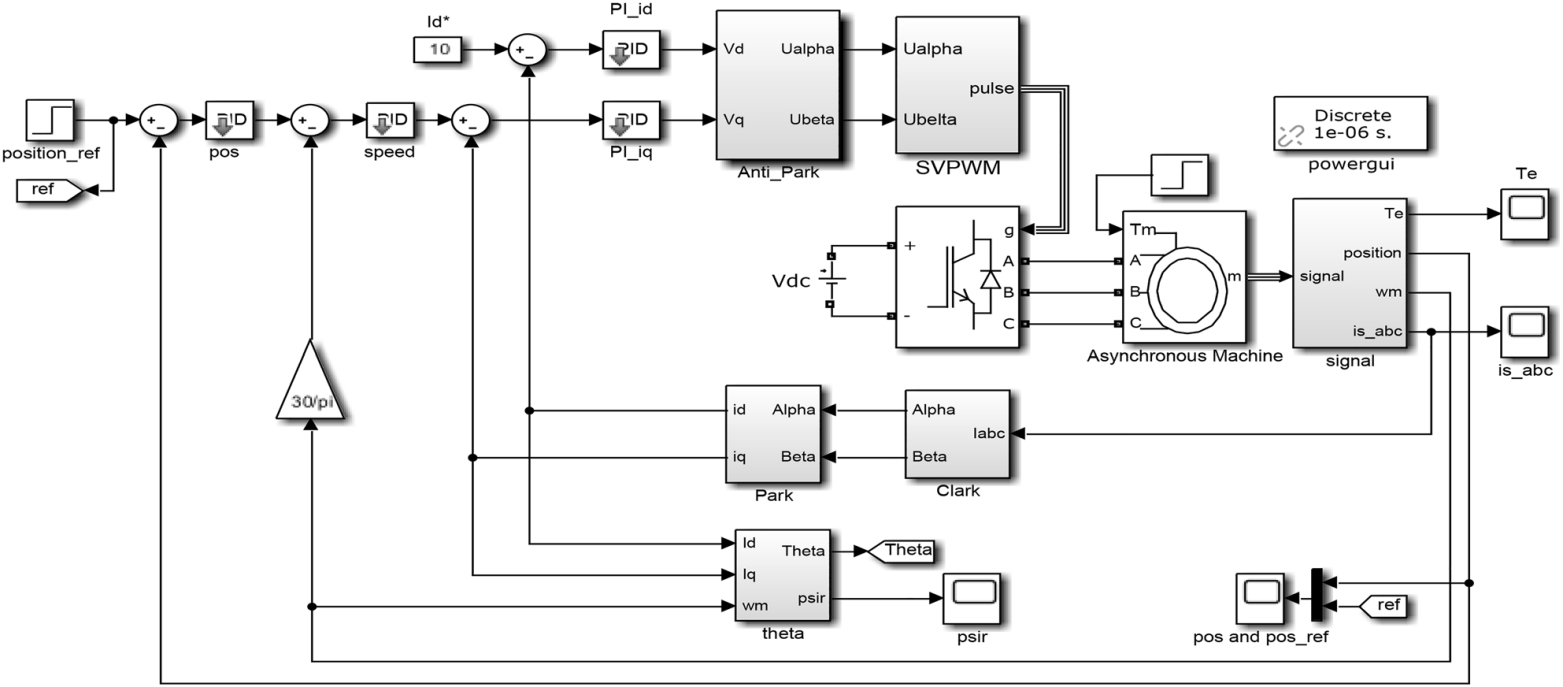
Simulink simulation model diagram of the control system.

**Figure 9:**
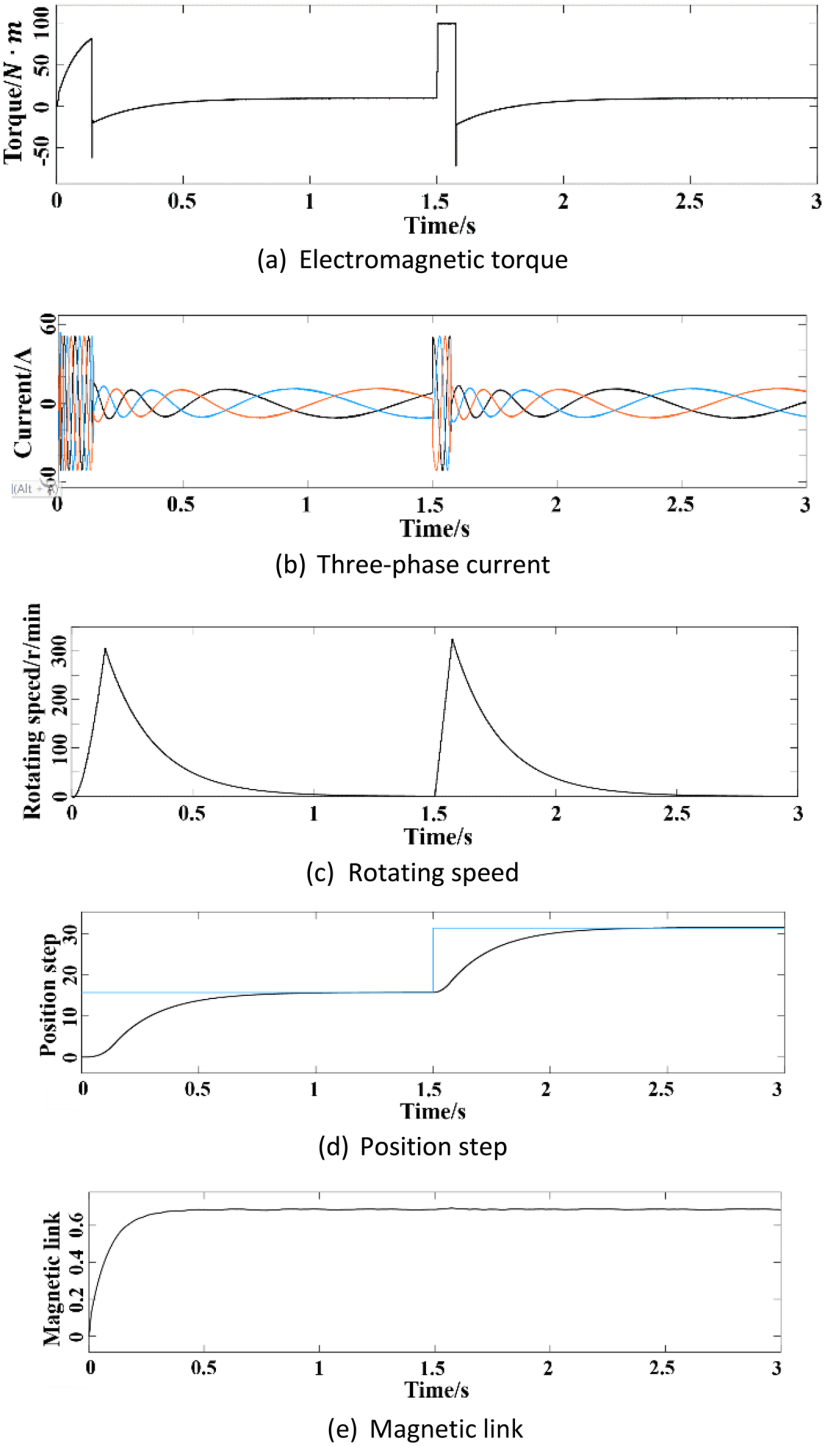
Simulation results of the control system performance under different operating conditions.

The purpose of adopting three closed-loop control of position, speed and current is to track quickly when the position coordinate or torque value changes, and to make the tracking performance good. The Simulink simulation model of the control system is shown in Fig. 8. Figure 9 shows the simulation results of the control system performance under different operating conditions. It can be seen from Fig. 9a and c that under the square wave signal input, when the motor is started without load and the motor accelerates, a large torque and speed are required. After the load is added, the torque and speed recover quickly in a short time.

It can be seen from the three-phase current change curve in Fig. 9b that the motor needs a large current when it starts to accelerate, and the current can return to normal after the load is added. It can be seen from Fig. 9d that the motor can reach the designated position smoothly with almost no overshoot. When the motor load changes suddenly, the dynamic response effect is good, and the new position can be tracked. The flux linkage change curve in Fig. 9e shows that the flux linkage can still be effectively tracked when the motor load changes suddenly, indicating that the dynamic performance of the control system is better.

Overall, the motor can reach a steady state in a short period of time after starting. After adding load disturbance, although the waveform has small fluctuations, it can quickly return to stability. Each output can also adapt to the change of sudden load in time, fully meeting the control requirements.

### Experimental test of control system performance

The control system of the building material packaging unit designed in this paper is controlled by the Wei-Lun-Tong touch screen. The man–machine interface uses modular programming method. By assigning addresses and data to the corresponding modules, the touch screen online simulation and compiling and downloading are completed. The man–machine interface has the characteristics of easy operation and information visualization, and the information transmission between the user and the operating system can be conveniently carried out^44,45^. Through the man–machine interface, users can efficiently modify and debug the parameters of the control system, and the relevant historical data of PLC can also be queried through the man–machine interface.

**Figure 10:**
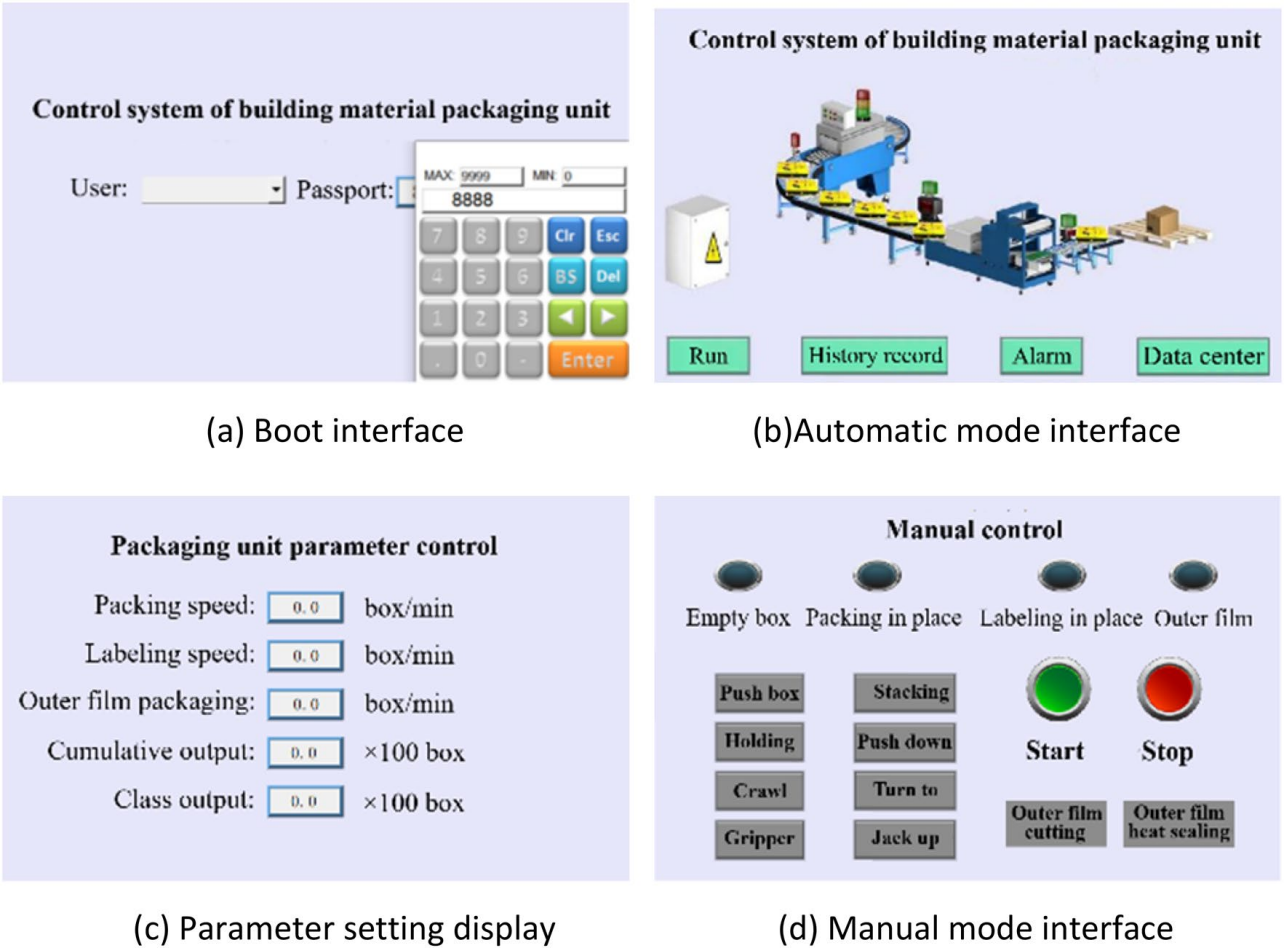
Touch screen interface design.

According to the design requirements, it is necessary to design the interfaces corresponding to the automatic and manual working modes. The manual interface includes the jog operation and step operation of each pneumatic actuator. In order to prevent mis-operation by non-professional operators, a user login password is set in the setting interface, as shown in Fig. 10a. Figure 10b and d show the automatic operation interface and manual operation interface. After confirming the setting of relevant parameters such as speed and output (seen in Fig. 10c), the system can enter the fully automatic operation or manual debugging mode interface.

After the simulation is completed, in order to further verify the feasibility of the packaging unit, the on-site operation and debugging of the packaging unit system is carried out based on the touch screen interface. Table 4 shows the experimental performance indicators of the building material packaging automatic control system. The fast recovery of the position can be observed within 1 s no matter in the no-load or loaded state. After the motor starts, although the steady-state error in the load state is slightly higher than that in the no-load state, it can still reach the steady state in a short time. The torque and speed under load state are slightly higher than those under no-load state, and each output can adapt to the change of sudden load in time.

**Table 4:** Experimental performance index of control system.

Working status	Steady-state error	Adjustment time $\left({\varvec{s}}\right)$	Torque $\left(N\cdot m\right)$	Rotating speed $\left(r\cdot {min}^{-1}\right)$	position recovery time $\left({\varvec{s}}\right)$
No loaded	0.013	0.274	79.18	305.83	0.87
Load	0.076	0.206	101.02	342.01	0.57

Table 5 shows the running speed field test data recorded by multiple tests. It can be seen from the test data that the operating rate of the whole machine is 98.15%, indicating that the mechanical structures and electrical control components can operate in harmony; after a certain period of continuous operation, the production capacity of the packaging unit can reach about 9600 boxes/h, which meets the needs of industrial production. The rated production capacity has improved the production efficiency, and the qualified rate has reached 99.03%, within a reasonable range, the packaging quality is basically qualified, and the control effect is good. The test analysis shows that the operation of the designed carton packaging unit is consistent with the requirements, which fully verifies the effectiveness of the control system.

**Table 5:** Production capacity and production efficiency of packaging unit system.

Number of trials	Packing time $\left(s\right)$	Pass rate $\left(\boldsymbol{\%}\right)$	Production capacity $(boxes/h)$	Overall effective operation rate $\left(\boldsymbol{\%}\right)$
1	62	99.5	9600	97.6
2	59	98.9	9580	98.2
3	60	99.1	9600	98.5
4	65	99.6	9650	99.4
5	61	99.3	9610	98.7
6	58	97.8	9570	96.5
Average	60.83	99.03	9601.67	98.15
Ref^46^	60	89.85	6296	89.85
Ref^47^	60	98.46	9438.90	98.46

In order to further verify the contribution and advantages of the automatic packaging control system in this study, we further compared the performance of the automatic packaging control system designed by other researchers in terms of pass rate, production capacity, and overall effective operation rate. It should be emphasized that since this article is the first study on the automatic packaging system of special building materials (no other similar systems were found through WOS and Google databases), we chose similar automatic packaging systems such as hard cigarette boxes and automatic bagging of cement packaging. Packaging systems for comparison. Obviously, from the comparison results in Table 5, it can be seen that the automatic packaging system for building materials in this article is much higher than the automatic packaging system for hard cigarette boxes in terms of pass rate, production capacity, and overall effective operation rate^46^. Compared with the cement packaging automatic bagging and packaging system, the system performance between the two maintains almost the same advantages^47^. However, it should be noted that the packaging process of cartons is far more complicated than bagging. Therefore, overall, the automatic packaging system for special building materials in this article has better performance, with the pass rate, production capacity and overall effective operation rate reaching 99.03%, 9600 boxes/h and 98.15% respectively. Obviously, multi-module coupling based on risk model, PLC control, PID control and AC servo system drive control has effectively improved the performance of the automatic packaging system for special building materials.

### Multi-field applicability and sustainability analysis of automatic packaging system

The technology and principles of automatic packaging systems for building materials also have potential application value in other industrial fields, such as food and beverages, medical equipment, hard-packed cigarettes, electronic products, etc. Servo motors, stepper motors, PLC control and other modules are very similar to other product packaging systems. If they are improved according to the packaging size and control requirements, they can be fully adapted to the automatic packaging of other items. Machine vision technology and automated assembly equipment can help realize automatic packaging and detection of food and beverage products, automatic packaging and assembly of medical devices, automatic assembly of electronic products, etc.^48,49^.

In the current industrial environment, our automated packaging systems for building materials strive to improve packaging efficiency while also paying attention to environmental impact and sustainability. The following are the considerations and advantages of our system in this regard:

1. Resource utilization efficiency: The system reduces material waste and loss and improves resource utilization efficiency through automated and precise packaging processes.
2. Energy saving: Due to efficient automated workflow, the system can reduce energy consumption, thereby reducing negative impact on the environment.
3. Reduce human errors: Automated systems reduce human errors and losses, reduce the production of substandard products, and are conducive to environmental protection and sustainability.
4. Sustainable packaging material selection: We encourage the use of sustainable packaging materials, and the system can adapt to multiple types of packaging materials, thereby reducing the impact on the environment.

These considerations and advantages reflect our system's focus on environmental impact and sustainability in the current industrial environment, and are committed to reducing resource waste and environmental burden.

### Case study

This case study is a secondary validation of the feasibility and practicality of the PID risk control model simulation using a new type of foam (EPP) as packaging material.

#### Project background

The investment estimate of the project for the production and construction of new type of foam plastic (EPP) products in Muding County is 120 million RMB. The project is applied in the fields of industry, construction and insulation packaging. the EPP is light in quality, which can reduce the weight of items substantially and can be recycled.

#### Case simulation

**Figure 11:**
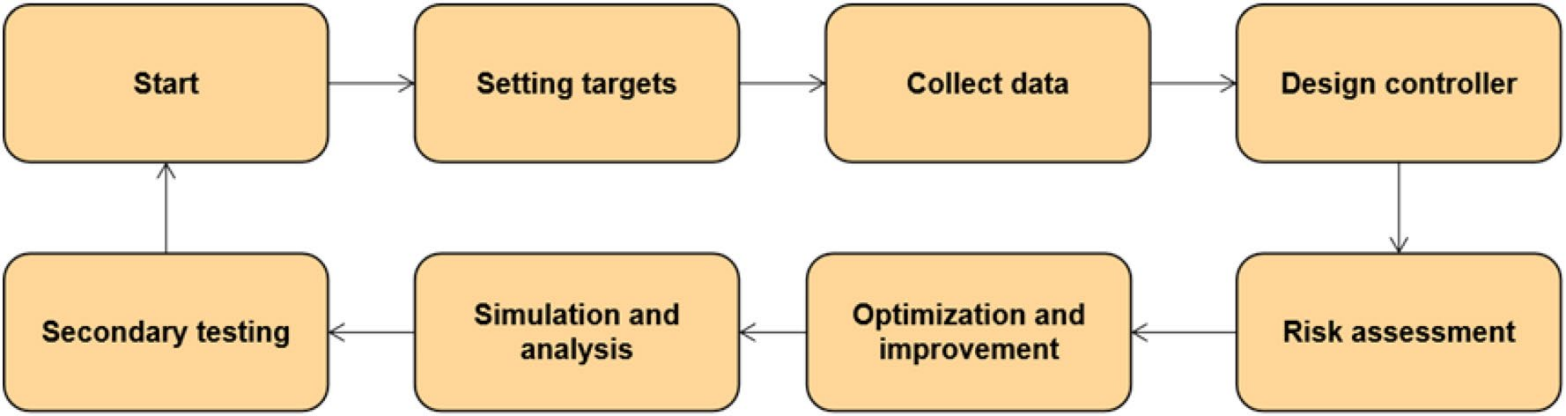
Flowchart of a case study in the packaging unit.

We can find that the building material packaging unit is completed according to the process shown in Fig. 11.

1. Setting targets: According to the quality and risk control requirements of construction material packaging, set the target value of risk control level as low risk. It also requires risk identification and assessment.
2. Collect data: Install sensors to monitor the operation status and packaging quality of construction material packaging machinery in real time, including personnel, equipment, monitoring and maintenance.
3. Design controller: PID controller can calculate the control quantity according to the real-time error signal, and transfer it to the construction material packaging machinery to adjust the parameters in the packaging process to achieve the control target.
4. Risk assessment: The risk assessment model is combined with the construction material packaging control model to analyze the risk situation of the construction material packaging machinery in real time, and dynamically adjust the parameters of the PID controller according to the changes in the degree of risk in order to achieve risk control.
5. Simulation and analysis: The construction material packaging control model is simulated and analyzed using Matlab and Python, and the stability and accuracy of the PID control model, as well as the control effect on the risk are verified.
6. Optimization and improvement: Based on the results of the simulation analysis, the PID control model is optimized and improved, including the updating of risk factors and the adjustment of the parameters of the PID controller in order to ensure the risk control of the construction material packaging unit.

## Conclusion and perspectives

This paper completes the risk assessment based on LEC evaluation, risk index matrix and CVaR model. According to the design of the control system of the building material packaging unit from the perspective of personnel, equipment, monitoring and maintenance risks, the automation and intelligent control of the production process of the packaging process has been completed. Using networked and intelligent technology, the information collected by the sensor on the field equipment layer and the PLC control layer are transmitted to each other, and the signal is received by the host computer through Ethernet, and the information layer man–machine interface intelligently monitors and manages the production process. It can not only alarm in time for the faults that occur during the operation process, but also set the operating speed and count the box output of the cumulative shift, which fully meets the process and control requirements.

Since the packaging unit has certain requirements on the stability and positioning accuracy of the control system, a mathematical model of the servo control system was established and simulated with Simulink based on the PID control model, which is mainly used to optimize parameters such as speed and position. The comparison of simulation results shows that under different working conditions, the control system has strong anti-disturbance ability and high steady-state accuracy, and has good robustness. Finally, the on-site experiment of building material packaging was further carried out. During the experiment, the packaging unit can effectively and smoothly run from boxing, labeling to outer film packaging, and the production capacity and pass rate are as high as 97%, which verifies the reliability of the control system. The packaging unit control system designed in this paper combines the risk management model, and lays the foundation for the design of the mechatronics program for building material packaging production, and provides a certain reference and promotion value for intelligent control in other industries.

## Data Availability

The data that supports the findings of this study are available within the article.
